# Estimating the prevalence, factors, and conditions associated with Parkinson disease: a population-based study in Peru

**DOI:** 10.1590/0102-311XEN011324

**Published:** 2024-09-20

**Authors:** Antonio Bernabe-Ortiz, Rodrigo M. Carrillo-Larco

**Affiliations:** 1 Universidad Científica del Sur, Lima, Perú.; 2 Rollins School of Public Health, Emory University, Atlanta, U.S.A.

**Keywords:** Parkinson Disease, Depression, Anxiety, Cognitive Dysfunction, Quality of Life, Enfermedad de Parkinson, Depresión, Ansiedad, Disfunción Cognitiva, Calidad de Vida, Doença de Parkinson, Depressão, Ansiedade, Disfunção Cognitiva, Qualidade de Vida

## Abstract

This study aimed to estimate the population-based Parkinson disease prevalence, and to explore potentially associated factors and conditions. A population-based survey was conducted in Northern Peru. Symptoms compatible with Parkinson’s were defined using a validated Spanish questionnaire (≥ 42 points suggest Parkinson’s). Potential factors (e.g., age, sex, etc.) and clinical conditions (e.g., depressive symptoms, perceived stress, etc.) associated with Parkinson’s were assessed. In total, 1,609 subjects were included, mean age of participants was 48.2 (SD: 10.6), and 810 (50.3%) were women. Parkinson’s prevalence was 1.6% (95%CI: 1.0; 2.4). Those aged ≥ 55 years, and those who reported using wood as fuel for household cooking had a Parkinson’s prevalence from 3.5 to 4 times greater than those who did not. The presence of depressive symptoms, anxiety symptoms, perceived stress, poor sleep quality, and cognitive impairment was more common among those with Parkinson’s, and quality of life in these participants was lower than those without Parkinson’s. In conclusion, 1.6% of the population shows symptoms compatible with Parkinson’s. Age and use of wood for household cooking were factors associated with Parkinson’s. Several mental health conditions and lower quality of life were more frequent among those with Parkinson’s. Appropriate strategies are required to detect, prevent, and manage Parkinson’s cases.

## Resumen

Este estudio tuvo como objetivo estimar la prevalencia poblacional de la enfermedad de Parkinson y explorar los factores y condiciones potencialmente asociados. Se realizó una encuesta con base poblacional en el norte del Perú. Los síntomas consistentes con la enfermedad de Parkinson se definieron a partir de un cuestionario validado en español (≥ 42 puntos sugieren enfermedad de Parkinson). Se evaluaron los factores potenciales (edad, sexo, etc.) y el estado clínico (síntomas depresivos, estrés percibido, etc.) asociados con la enfermedad de Parkinson. Participaron 1.609 personas, de edad media de 48,2 (DE: 10,6) y 810 (50,3%) participantes eran del sexo feminino. La prevalencia de la enfermedad fue del 1,6% (IC95%: 1,0; 2,4). Los participantes de edad superior a 55 años y los que usaban leña para cocinar tuvieron una prevalencia de 3,5 a 4 veces mayor de presentar enfermedad de Parkinson en comparación con aquellos que no lo hicieron. La presencia de síntomas depresivos, ansiedad, estrés percibido, mala calidad del sueño y deterioro cognitivo fueron más frecuentes entre aquellos con enfermedad de Parkinson; y la calidad de vida en estos participantes fue menor en comparación con la de las personas sin enfermedad de Parkinson. Se concluye que el 1,6% de esta población presenta síntomas compatibles con la enfermedad de Parkinson. La edad y el uso de leña para cocinar fueron factores asociados a enfermedad de Parkinson. Diversas condiciones de salud mental y menor calidad de vida fueron más frecuentes entre las personas con enfermedad de Parkinson. Se necesitan estrategias específicas para el diagnóstico, prevención y tratamiento de los casos de enfermedad de Parkinson.

## Resumo

Este estudo teve como objetivo estimar a prevalência populacional da doença de Parkinson e explorar fatores e condições potencialmente associados. Uma pesquisa de base populacional foi realizada no norte do Peru. Os sintomas compatíveis com doença de Parkinson foram definidos por meio de um questionário em espanhol validado (≥ 42 pontos sugerem doença de Parkinson). Foram avaliados fatores potenciais (p.ex.:, idade, sexo etc.) e condições clínicas (p.ex.: sintomas depressivos, estresse percebido etc.) associados à doença de Parkinson. No total, foram incluídos 1.609 sujeitos, a média de idade dos participantes foi de 48,2 (DP: 10,6) e 810 (50,3%) eram mulheres. A prevalência da doença foi de 1,6% (IC95%: 1,0; 2,4). Aqueles com idade ≥ 55 anos e aqueles que relataram usar lenha como combustível para cozinhar em casa tiveram prevalência de doença de Parkinson de 3,5 a 4 vezes maior do que aqueles que não. A presença de sintomas depressivos, sintomas de ansiedade, estresse percebido, má qualidade do sono e comprometimento cognitivo foram os mais comuns entre aqueles com doença de Parkinson, e a qualidade de vida nesses participantes foi menor do que a daqueles sem doença de Parkinson. Em conclusão, 1,6% da população apresenta sintomas compatíveis com doença de Parkinson. A idade e o uso de lenha para cozinhar em casa foram fatores associados à doença de Parkinson. Várias condições de saúde mental e menor qualidade de vida foram mais frequentes entre aqueles com doença de Parkinson. Estratégias apropriadas são necessárias para detectar, prevenir e gerenciar casos de doença de Parkinson.

## Introduction

Globally, Parkinson disease is one of the most common neurodegenerative conditions, characterized by progressive motor symptoms ^1^. According to the *Global Burden of Disease Study* (GBD), an increase in the number of Parkinson’s cases has been observed worldwide since 1990 ^2^. In women, the incidence rate has increased from 3.3 at the age of 40-49 years to 103.5 per 100,000 people-year in those ≥ 80 years of age; whereas in men, these estimates were 3.6 and 258.5 per 100,000 person-years, respectively ^3^. In Peru, the prevalence of Parkinson’s has been estimated being greater among females (1.8%) than males (0.9%) aged 65 years and over ^4^.

As the aging process affects the general population, it is mandatory to determine the prevalence, as well as the factors and conditions associated with certain neurodegenerative conditions, especially in resource-constrained settings. Thus, such information can serve to inform policies, interventions, and priorities, as well as patient screening. Estimating common epidemiological indicators (e.g., prevalence) of these conditions is one of the major issues due to the difficulty of performing the diagnosis, as a structured interview is usually required, as well as physical and neurological examinations performed by specialists. However, several tools and scales can aid estimate the prevalence of motor symptoms of Parkinson’s and, thus, provide preliminary evidence on the prevalence of Parkinson’s in the general population ^5^ ^,^ ^6^ ^,^ ^7^.

Age and schooling level have been associated with the presence of Parkinson’s ^8^ ^,^ ^9^ ^,^ ^10^; nevertheless, few studies have evaluated the associated factors in low-income countries, which present different sociodemographic and epidemiological profiles, as well as determinants of access to health. Recently, a report using information from the *10/66 Study* ^4^, conducted with people aged 65 years and older from urban and rural areas, reported that Parkinson’s was more frequent at older ages and in those with a lower schooling level. On the other hand, other studies have focused on conditions that usually occur in those with Parkinson’s, such as mental health conditions, including depression ^11^ and cognitive impairment (dementia) ^4^. Evaluating different aspects of mental health and quality of life in these patients becomes a priority to understand the multimorbidity profile that they have. Therefore, people with Parkinson’s can be approached holistically, considering their entire health profile, rather than only Parkinson’s.

As a result, the main aim of this study was twofold: (i) to determine the prevalence of symptoms compatible with Parkinson’s at the population level, and (ii) to explore the potential factors and conditions associated with these symptoms, reanalyzing the data from a population study carried out in the north of Peru.

## Methods

### Design and study location

This is a secondary data analysis using a study to evaluate screening methods for type 2 diabetes mellitus at the population level ^12^. Fieldwork activities were conducted in the periurban area of Tumbes, in Northern Peru, during December 2016 and November 2017.

### Study population and sampling

Selection criteria of the original study included men and women, aged 30 to 69 years, habitual residents living ≥ 6 months in the study area, and capable of signing an informed consent form to participate. Pregnant patients at the moment of the study, as well as those bedridden or with physical disabilities precluding anthropometric measurements, were excluded. For this analysis, only those with incomplete information in the Parkinson’s evaluation were not included.

Participants were selected from the general population, regardless of any previous condition before selection. Sampling was conducted using a sex-stratified random approach, and study participants were selected using the most current census of the area (2014). Only one participant per household was enrolled in the study to avoid clustering of common risk factors.

Since this is a secondary analysis, with 1,600 participants, we will be able to detect a Parkinson’s prevalence of 1.6% with a precision of 0.7% and a 95% confidence level.

### Study variables

The variable of interest was the presence of symptoms compatible with Parkinson’s, obtained using the 9-item screening questionnaire developed by Tanner symptom questionnaire ^13^, translated and adapted by Duarte et al. in Spain ^5^. The instrument comprises nine questions that can be administered by a trained interviewer or physician or self-administered. In answering this instrument, participants have two potential responses (yes/no), which are weighted considering the common deficits associated with Parkinson’s to improve the specificity of the instrument. Thus, the score can range from 0 to 67, and it is considered positive (that is, it suggests Parkinson’s) in those with ≥ 42 points. With this cut-off point, the sensitivity and specificity of the instrument has been described as close to 100% ^5^.

Potential factors associated with Parkinson’s and reported in literature were included: sex (man vs. women), age group (< 40, 40-54, and 55+ years), schooling level (primary [< 7 years], secondary [7-11 years], and tertiary [12+ years]), socioeconomic status (based on household possessions and categorized into tertiles), use of firewood/wood as fuel for household cooking (rather than propane gas, yes/no), history of tobacco use (never/ever in life), alcohol consumption (never/ever in life), physical activity levels (based on the *International Physical Activity Questionnaire - Short Form* [IPAQ-SF], and classified as low/moderate or high), body mass index (normal, overweight, and obesity, defined based on international standards), hypertension (defined based on blood pressure measurements [systolic ≥ 140mmHg or diastolic ≥ 90mmHg] or previous self-reported medical diagnosis) ^14^, and type 2 diabetes mellitus (defined according to the oral glucose tolerance test [fasting glucose ≥ 126mg/dL or postprandial glucose ≥ 200mg/dL] or previous medical diagnosis) ^15^.

Mental health conditions were evaluated as potential factors associated with Parkinson’s. The presence of depressive symptoms was evaluated using the *Patient Health Questionnaire-9* (PHQ-9), a 9-item instrument with four response options ranging from “not at all” to “nearly every day”; a score ≥ 5 was defined as a cut-off point, compatible with the presence of mild depressive symptoms ^16^ ^,^ ^17^. The presence of anxiety symptoms was assessed using the *Goldberg Anxiety Scale* (GAS), a 9-item instrument that allows dichotomous answers (yes/no); a score ≥ 4 was defined as a cut-off point for the presence of symptoms compatible with anxiety ^18^. Perceived stress was assessed using the *Perceived Stress Scale* (PSS), a 14-item tool to rate feelings and thoughts about life events using a 5-point Likert type scale, ranging from “never” to “very often” ^19^. For this analysis, the level of perceived stress was divided into tertiles (low, moderate, and high) and compared considering the high-level category versus the grouped low- and moderate-level categories. Sleep quality was assessed using the *Pittsburgh Sleep Quality Index* ^20^, a 21-question instrument (subjective sleep quality, duration, sleep disturbances, latency, use of sleep medication, efficiency, and nocturnal dysfunction). The global score ranges from 0 to 21 points, in which > 5 points is considered a cut-off point for poor sleep quality. Cognitive impairment was assessed using the *Leganés Cognitive Test* (LST) ^21^ with a cut-off point of < 26. The test consists of 32 items that mainly evaluate memory, orientation, and attention. Finally, quality of life was assessed using the *EuroQoL-5 Dimension* (EQ-5D) ^22^, a questionnaire that includes a visual analogue scale to determine perceived quality of life by asking participants to classify their current state of well-being from zero, the worst health imaginable, to 100, the best health imaginable.

### Procedures

Data collection was conducted using tablet devices and the Open Data Kit (https://opendatakit.org/software/), an open-source mobile data collection platform that allows users to complete and save offline forms and then upload data to a server when Internet connection is available.

Fieldworkers were trained to administer the questionnaire and perform anthropometric measurements. Weight and height, to estimate body mass index (BMI), were obtained using standard techniques. Blood pressure was measured in triplicate using an automatic monitor (HEM-780, OMRON; https://www.omron.com/global/en/), collected after five minutes of resting with at least one minute between measurements. The average of the second and third measurements were used for analysis ^14^.

The glucose tolerance test was performed using international standards, with an 75g anhydrous glucose load and blood samples taken after 8-12 hours of fasting, and 2 hours postprandial ^15^. Forms and questionnaires, as well as body measurements, were administered and performed between sample collection for glucose tolerance.

### Statistical analysis

Analyses were performed using the Stata statistical software, version 16 (https://www.stata.com). Data were described using means and standard deviations (SD) for numerical variables, and absolute and relative frequencies for categorical variables. Cronbach’s alpha was used to evaluate the reliability of the instrument used to evaluate Parkinson’s, using 0.70 as a cut-off point to be considered acceptable ^23^. The prevalence and 95% confidence intervals (95%CI) of variables of interest were reported and, considering the limited number of events, differences in the distribution of Parkinson’s frequency according to the covariates were estimated using the Fisher’s exact test.

To explore the factors associated with Parkinson’s, Poisson regression models with robust variance were used. Given the exploratory nature of this analysis, in which Parkinson’s was the dependent variable, the multivariable model included all the studied variables according to the literature and that were available in the database, reporting prevalence ratios (PR) and 95%CIs.

Furthermore, to evaluate the conditions associated with Parkinson’s (considered in this case as an exposure variable), Poisson regression models were also built, both crude and adjusted but, in this case, the multivariable models were adjusted only for the most relevant confounding factors, that is, age, sex, schooling level, socioeconomic level, and PRs and 95%CIs were reported.

Regarding quality of life, given that it is a numerical variable, linear regression models with robust variance were used and coefficients (β) and 95%CIs were estimated. In all cases, a value of p < 0.05 was considered statistically significant.

### Ethical aspects

The study protocol and informed consent forms were approved by the Research Ethics Committees of the Cayetano Heredia Peruvian University (Universidad Peruana Cayetano Heredia, Peru) and the London School of Hygiene and Tropical Medicine (United Kingdom). Written informed consent was applied before commencing study activities. The database was de-identified to guarantee the anonymity of the participants.

## Results

### Characteristics of the study population

A total of 2,114 subjects were invited to the study, and of them, 486 (22.9%) refused to participate, whereas 16 (0.8%) women were pregnant at the time of the study and were excluded. Of the 1,612 (76.3%) subjects enrolled, three did not complete all phases of the study and, thus, 1,609 participants were analyzed in the present work. Mean age of participants was 48.2 years (SD: 10.6), 810 (50.3%) were women, and 519 (32.3%) had at least primary education.

### Prevalence of Parkinson disease and associated factors

The reliability of the 9-item questionnaire used to assess Parkinson’s was in an acceptable range (Cronbach’s α = 0.76). In total, 26 (1.6%; 95%CI: 1.0; 2.4) subjects presented symptoms and scores compatible with Parkinson’s. This condition was more frequent among females, those 55+ years old, with a lower schooling level, those who reported using wood as fuel for household cooking, and in those with obesity, hypertension, and type 2 diabetes mellitus (Table 1).

**Table 1:** Characteristics of the study population according to the presence of symptoms compatible with Parkinson disease, Peru.

Characteristics	Parkinson disease		p-value *
	No (n = 1,583)	Yes (n = 26)	
	n (%)	n (%)	
Sex			0.020
Male	792 (99.1)	7 (0.9)	
Female	791 (97.6)	19 (2.4)	
Age (years)			0.001
< 40	439 (99.6)	2 (0.4)	
40-54	681 (98.8)	8 (1.2)	
55+	463 (96.7)	16 (3.3)	
Schooling level			0.001
Primary	502 (96.7)	17 (3.3)	
Secondary	743 (99.2)	6 (0.8)	
Higher education	338 (99.1)	3 (0.9)	
Socioeconomic status			0.340
Low tertile	529 (98.0)	11 (2.0)	
Middle tertile	540 (98.2)	10 (1.8)	
High tertile	514 (99.0)	5 (1.0)	
Use of wood for household cooking			0.001
No	1,475 (98.7)	20 (1.3)	
Yes	108 (94.7)	6 (5.3)	
History of tobacco use			0.110
Never	972 (98.0)	20 (2.0)	
Ever in life	611 (99.0)	6 (1.0)	
Alcohol consumption			0.120
Never	671 (97.8)	15 (2.2)	
Ever in life	912 (98.8)	11 (1.2)	
Physical activity levels			0.360
Moderate/High	990 (98.6)	14 (1.4)	
Low	593 (98.0)	12 (2.0)	
Body mass index			0.020
Normal	419 (98.6)	6 (1.4)	
Overweight	702 (99.2)	6 (0.9)	
Obesity	462 (97.1)	14 (2.9)	
Hypertension			0.005
No	1,179 (98.9)	13 (1.1)	
Yes	404 (96.9)	13 (3.1)	
Type 2 diabetes mellitus			0.009
No	1,412 (98.7)	19 (1.3)	
Yes	169 (96.0)	7 (4.0)	

* p-value was estimated using Fisher’s exact test.

In the multivariable model, those aged 55+ years (PR = 4.05; 95%CI: 1.01; 16.34) and those who reported using wood as fuel for household cooking (PR = 3.45; 95%CI: 1.25; 9.48) were most likely to have symptoms compatible with Parkinson’s (Table 2).

**Table 2:** Factors associated with symptoms compatible with Parkinson disease in Peru: bivariable and multivariable models.

Characteristics	Bivariable model	Multivariable model *
	PR (95%CI)	PR (95%CI)
Sex		
Male	1.00 (Reference)	1.00 (Reference)
Female	2.68 (1.13; 6.34)	2.14 (0.58; 7.93)
Age (years)		
< 40	1.00 (Reference)	1.00 (Reference)
40-54	2.56 (0.55; 12.01)	1.87 (0.45; 7.75)
55+	7.37 (1.70; 31.90)	4.05 (1.01; 16.34)
Schooling level		
Primary	1.00 (Reference)	1.00 (Reference)
Secondary	0.24 (0.10; 0.62)	0.55 (0.19; 1.62)
Higher education	0.27 (0.08; 0.91)	0.72 (0.22; 2.36)
Socioeconomic status		
Low tertile	1.00 (Reference)	1.00 (Reference)
Middle tertile	0.89 (0.38; 2.08)	1.22 (0.47; 3.17)
High tertile	0.47 (0.17; 1.35)	0.95 (0.28; 3.26)
Use of wood for cooking		
No	1.00 (Reference)	1.00 (Reference)
Yes	3.93 (1.61; 9.60)	3.45 (1.25; 9.48)
History of tobacco use		
Never	1.00 (Reference)	1.00 (Reference)
Ever in life	0.48 (0.19; 1.19)	0.78 (0.27; 2.26)
Alcohol consumption		
Never	1.00 (Reference)	1.00 (Reference)
Ever in life	0.55 (0.25; 1.18)	1.44 (0.55; 3.76)
Physical activity levels		
Moderate/High	1.00 (Reference)	1.00 (Reference)
Low	1.42 (0.66; 3.06)	1.03 (0.46; 2.32)
Body mass index		
Normal	1.00 (Reference)	1.00 (Reference)
Overweight	0.60 (0.19; 1.85)	0.53 (0.17; 1.60)
Obesity	2.08 (0.81; 5.37)	1.73 (0.66; 4.49)
Hypertension		
No	1.00 (Reference)	1.00 (Reference)
Yes	2.86 (1.34; 6.12)	1.89 (0.95; 3.77)
Type 2 diabetes mellitus		
No	1.00 (Reference)	1.00 (Reference)
Yes	3.00 (1.28; 7.03)	1.86 (0.74; 4.63)

95%CI: 95% confidence interval; PR: prevalence ratio.

* The model is adjusted for all the variables listed.

### Conditions associated with Parkinson disease

Mental health and clinical conditions evaluated were significantly associated with the presence of Parkinson’s (Table 3). In a multivariable model of patients with Parkinson’s, adjusted for age, sex, schooling level, and socioeconomic level, the presence of Parkinson’s was associated with significant increases in various mental health conditions compared to those without Parkinson’s. Specifically, the presence of Parkinson’s was linked to a 181% increase (95%CI: 115; 269) in the prevalence of depressive symptoms, a 100% increase (95%CI: 71; 133) in anxiety symptoms, a 140% increase (95%CI: 93; 199) in high perceived stress, a 140% increase (95%CI: 66; 246) in poor sleep quality, and a 93% (95%CI: 3; 262) increase in cognitive impairment. Finally, participants with symptoms compatible with Parkinson’s had a quality of life 15.6 (95%CI: 8.0; 23.3) points lower than those without those symptoms.

**Table 3:** Conditions associated with symptoms compatible with Parkinson disease in Peru: crude and adjusted models.

Conditons	Parkinson disease		Crude model	Adjusted model *
	No (n = 1,583)	Yes (n = 26)	PR (95%CI)	PR (95%CI)
	n (%)	n (%)		
Depressive symptoms				
No	1,229 (77.6)	6 (23.1)	1.00 (Reference)	1.00 (Reference)
Yes	354 (22.4)	20 (76.9)	3.44 (2.73; 4.33)	2.81 (2.15; 3.69)
Anxiety symptoms				
No	932 (58.9)	2 (7.7)	1.00 (Reference)	1.00 (Reference)
Yes	651 (41.1)	24 (92.3)	2.24 (1.98; 2.55)	2.00 (1.71; 2.33)
Perceived stress				
Low/Moderate	1,099 (69.4)	4 (15.4)	1.00 (Reference)	1.00 (Reference)
High	484 (30.6)	22 (84.6)	2.77 (2.31; 3.31)	2.40 (1.93; 2.99)
Sleep quality				
Good	1,283 (81.2)	11 (42.3)	1.00 (Reference)	1.00 (Reference)
Poor	297 (18.8)	15 (57.7)	3.07 (2.17; 4.33)	2.40 (1.66; 3.46)
Cognitive impairment				
No	1,394 (88.1)	18 (69.2)	1.00 (Reference)	1.00 (Reference)
Yes	189 (11.9)	8 (30.8)	2.58 (1.43; 4.66)	1.93 (1.03; 3.62)
Quality of life ** [mean (SD)/coefficient (β)]	72.7 (16.4)	54.0 (19.6)	-18.6 (-26.10; -11.20)	-15.6 (-23.30; -8.00)

95%CI: 95% confidence interval; PR: prevalence ratio.

* Model adjusted for age, sex, schooling level, and socioeconomic status;

** Quality of life was assessed by comparison of means and using linear regression models.

## Discussion

### Main findings

This study shows that 1.6% of the general population in a periurban site in Northern Peru aged from 30 to 69 years had symptoms compatible with Parkinson’s, being higher in those ≥ 55 years of age. Moreover, the self-reported use of wood/firewood as fuel for household cooking was a factor significantly associated with Parkinson’s. Finally, several mental health conditions were more frequent among those with Parkinson’s, including symptoms of depression, anxiety, perceived stress, poor quality of sleep, cognitive impairment, and lower quality of life, compared to those without Parkinson’s.

### Comparison with previous studies

A recent systematic review about Parkinson’s in Latin America reported 0.47% prevalence using information from 17 studies ^24^; however, estimates differed by data source and age. According to a global study, more than 8 million individuals had Parkinson’s worldwide in 2019, with a relative percentage change greater than 150% compared to 1990 ^2^. In the same study, this value for Andean Latin America, where Peru is located, has been estimated at 54,000 subjects with Parkinson’s with an increase of 107% in the same period. The *10/66 Study*, which enrolled subjects ≥ 65 years of age, reported that Parkinson’s prevalence in the urban area of Lima was 7.9%, whereas in the rural area of Cañete (south of Lima) it was 6.6% ^4^. Although our results show lower estimates, the results were obtained via neurological tests and evaluations conducted by specialists in the *10/66 Study*, thus they were able to capture more cases compared to our study. Moreover, since age groups were different, our estimates showed lower results than those reported in the *10/66 Study*.

On the other hand, in other similar contexts such as Colombia, the age-adjusted prevalence of Parkinson’s was 0.3% in individuals aged 60-69 years, 1.1% in those aged 70-79 years, and 1.8% in those aged 80-89 years ^8^. Nevertheless, in this last study, Parkinson’s was defined based on a clinical diagnosis algorithm and pharmacological prescription of each subject. Thus, our results, based on a motor symptoms questionnaire for Parkinson’s, are within the expected values for this condition according to other studies in similar contexts.

Regarding the associated factors, as in other studies, older age was associated with a higher prevalence of Parkinson’s ^8^ ^,^ ^24^. Moreover, the self-reported use of firewood/wood as fuel for household cooking was strongly associated with the presence of Parkinson’s, even after controlling for different confounders. Tumbes, the area of study, is an arid and subtropical region located in Northern Peru, close to the border with Ecuador. Use of wood for household cooking is important, especially in rural and semiurban areas, where the study was conducted, but this activity is usually carried out outdoors, as weather temperature averages from 27ºC to 35ºC ^25^. According to the literature, certain pollutants, such as tobacco or exposure to pesticides, are consistently associated with higher prevalence of Parkinson’s, as well as environmental pollution ^26^. Long-term exposure to some metals, such as copper, manganese, mercury, zinc, and lead, have also been associated with higher Parkinson’s risk ^27^. Thus, although wood/firewood smoke could be one of these pollutants, other related chemicals, such as the type of wood used or contamination with pesticides, could be options to consider. For example, a study conducted in Louisiana, in the United States, using information from hospital primary discharge, reported that commercial forests, woodlands, and pastures - thus, some pastoral pesticides - may be associated with Parkinson’s ^28^. Agriculture and pesticide use (i.e., paraquat and methomyl 90SP) are common in Tumbes, and metal contamination of water with cadmium and arsenic has been described as well. Thus, this is a topic that requires further study using longitudinal studies.

Finally, several conditions, especially related to mental health and quality of life, were associated with Parkinson’s. In all cases, the presence of Parkinson’s at least doubles the prevalence of any mental health indicator, as the literature demonstrates in the case of depression ^11^ and anxiety ^29^. In the case of cognitive impairment, even the medication used, including levodopa, has been associated with cognition, both in deleterious ways and with beneficial effects, depending on the person’s susceptibility ^30^. Finally, regarding quality of life, a systematic review shows that people with Parkinson’s have lower quality of life compared to healthy controls in almost all domains, especially in physical and mental function ^31^, probably because people with Parkinson’s cannot, or need help, to perform basic daily tasks.

### Public health relevance

This study highlights several aspects of interest. First, the need for appropriate tools to detect and screen Parkinson’s at the population level. This is important since the literature has been demonstrating an aging process in the general population, which leads to the increase in degenerative diseases ^24^ ^,^ ^32^, including neurological conditions. If a single instrument appears to be insufficient to “diagnose” Parkinson’s or other neurodegenerative diseases, then an algorithm that combines several instruments and other variables should be designed. Second, determining, in greater detail, the factors that may favor the increase of Parkinson’s cases. In addition to age, the involvement of contaminants, such as wood for household cooking, must be explored longitudinally to reduce the onset of new cases. There is a possibility that contamination of firewood may be playing a role in the presence of Parkinson’s cases. Third, cases with Parkinson’s should be evaluated and treated appropriately for various mental health conditions. Finally, interventions are warranted to improve the quality of life of those suffering from Parkinson’s. This includes not only appropriate medication, but also comprehensive case management, such as management of sleep quality and stress, both for the patient and their close network, including family and caregiver. Similarly, the health and well-being of the close family of patients with Parkinson’s, particularly those who play the role of caregivers, must be studied and cared for.

### Strengths and limitations

This work benefits from using data from a population-based study in a specific area of Peru. We also highlight the use of a tool validated in Spanish to determine the outcome of interest. However, this work also shows limitations. First, due to the cross-sectional nature of the study, we cannot assess causality but only association. Although reverse causality and temporality could be concerns, it is unlikely that these exist in this work since several of the potential factors evaluated occurred for a long time prior to the development of Parkinson’s. For instance, household cooking with wood is probably the fuel they have used for many years and not recently adopted. Second, the employed questionnaire is based on the detection of motor symptoms of Parkinson’s (i.e., parkinsonism), which could underestimate the real prevalence of the disease since there are non-motor symptoms that can be highly frequent (about 90% of cases with Parkinson’s present non-motor symptoms) ^33^. On the other hand, the instrument used could not capture all cases of Parkinson’s due to the presence of diseases with similar symptoms. Moreover, this tool was designed for clinical settings and not for community use. Despite of that, the scale used has shown good diagnostic performance ^5^. Third, there could also be recall bias for some of the questions evaluated, especially those related to behaviors or lifestyles. In addition, pesticide use and other environmental factors associated with Parkinson’s were not evaluated in the original study, thus were not included in this manuscript. Fourth, the limited number of Parkinson’s cases detected in the study could hinder the precision of our estimates. However, our results corroborate previous studies. Finally, there may be selection bias since the chosen study area presents a higher prevalence of certain risk factors (type 2 diabetes mellitus, obesity, and hypertension), which could affect the generalizability of the results.

## Conclusions

We found that 1.6% of the general population aged from 30 to 69 years presented symptoms compatible with Parkinson’s. In addition to age, self-reported use of wood/firewood for household cooking was a factor strongly associated with Parkinson’s. Several mental health conditions and lower quality of life were more frequent in those with Parkinson’s when compared to those without Parkinson’s. Appropriate strategies are required for the detection and management of Parkinson’s cases.
